# Combining transcranial direct current stimulation with group cognitive behavioral therapy developed to treat rumination: a clinical pilot study

**DOI:** 10.3389/fneur.2023.1167029

**Published:** 2023-04-25

**Authors:** Paula Horczak, Chanyu Wang, Sara De Witte, Stefanie De Smet, Jonathan Remue, Rudi De Raedt, Marie-Anne Vanderhasselt, Guo-Rong Wu, Gilbert M. D. Lemmens, Chris Baeken

**Affiliations:** ^1^Ghent Experimental Psychiatry (GHEP) Lab, Ghent University, Ghent, Belgium; ^2^Department of Head and Skin – Psychiatry and Medical Psychology, Ghent University, Ghent, Belgium; ^3^Department of Neurology and Bru-BRAIN, University Hospital Brussels, Brussels, Belgium; ^4^Neuroprotection and Neuromodulation Research Group (NEUR), Center for Neurosciences (C4N), Vrije Universiteit Brussel (VUB), Brussels, Belgium; ^5^Department of Psychiatry, Universitair Ziekenhuis Brussel (UZ Brussel), Vrije Universiteit Brussel (VUB), Brussels, Belgium; ^6^Department of Psychiatry, Ghent University Hospital, Ghent, East Flanders, Belgium; ^7^Department of Experimental Clinical and Health Psychology, Ghent University, Ghent, Belgium; ^8^Key Laboratory of Cognition and Personality, Faculty of Psychology, Southwest University, Chongqing, China; ^9^Department of Electrical Engineering, Eindhoven University of Technology, Eindhoven, Netherlands

**Keywords:** transcranial direct current stimulation, group cognitive behavioral therapy, rumination, repetitive negative thinking, major depressive disorder, generalized anxiety disorder, NIBS, psychotherapy

## Abstract

**Background:**

As part of repetitive negative thinking (RNT), rumination is a maladaptive cognitive response style to stress or negative mood which can increase the risk of depression and may prohibit complete recovery. Cognitive behavioral therapy (CBT) and transcranial direct current stimulation (tDCS) both proved to be effective in decreasing rumination. However, the combined effects of tDCS and CBT interventions on rumination have not yet been explored. The first aim of this pilot study is to investigate whether the combination of tDCS and CBT has an accumulating positive effect on modulating state rumination. The second aim is to assess the feasibility and safety profile of the proposed combined approach.

**Method:**

Seventeen adults aged 32–60 years, suffering from RNT, were referred by their primary care professional to participate in an 8-week group intervention for RNT (“Drop It”) comprising 8 sessions of CBT. Before each CBT session, patients underwent one double-blinded prefrontal active (2 mA for 20 min) or sham tDCS (anode over F3, cathode over the right supraorbital region) combined with an internal cognitive attention task focused on individual RNT, i.e., online tDCS priming. During each session, the Brief State Rumination Inventory was used to assess state rumination.

**Results:**

A mixed effects model analysis revealed no significant differences between the stimulation conditions, weekly sessions, or their interaction in terms of state rumination scores.

**Conclusion:**

Overall, the combination of online tDCS priming followed by group CBT was found to be safe and feasible. On the other hand, no significant additional effects of this combined approach on state rumination were established. Although our pilot study may have been too small to find significant clinical effects, future larger RCT studies on combined tDCS-CBT treatment protocols may reevaluate the selection of internal cognitive attention tasks and more objective neurophysiological measurements, consider the optimal timing of the combination (concurrently or sequentially), or may add additional tDCS sessions when following CBT.

## Introduction

Repetitive negative thinking (RNT), such as rumination or worry, has been considered to be a transdiagnostic process for mood and anxiety disorders (1, 2). Rumination involves repetitively focusing on negative events and their potential causes and consequences and has been considered to be a maladaptive cognitive response style that can increase the risk of depression (3, 4). Worry, on the other hand, is more often associated with anxiety disorders, and is characterized by excessive and uncontrollable thoughts about potential negative outcomes or events in the future (5–7). Clinical management typically includes psychotherapy (8).

For instance, cognitive behavioral therapy (CBT) has been shown to be an effective treatment for reducing RNT in patients with major depressive disorder (MDD) and generalized anxiety disorder (GAD), this on the individual level but also when offered as group therapy (9, 10). In addition, it has been shown that RNT-focused CBT in particular has a more pronounced effect on RNT than treatments that do not specifically target rumination (11). For example, Watkins and colleagues (12) performed a randomized controlled trial which provided evidence that MDD patients in the rumination-focused CBT group ruminated significantly less compared to those in the treatment as usual group. Recently, Rogiers and colleagues (13) developed a psychoeducational CBT-based group intervention called “Drop It,” specifically for the treatment of RNT. It has proven to be effective in reducing RNT and improving the quality of life for MDD patients, which remain stable up to 9 months after the intervention (14). Brain imaging observations suggest its effectiveness in reducing RNT is associated with increased prefrontal brain perfusion in the left dorsolateral prefrontal cortex (DLPFC) (15). The DLPFC is implicated in regulating affective states and in providing cognitive control over stress and emotional responsiveness (16). In a healthy population, Kühn and colleagues (17) found that DLPFC activation and unwanted self-referent ruminative thoughts were inversely correlated. Crucially, as demonstrated by Jacobs and colleagues (18), adolescents at risk for depressive relapse, who received CBT, showed a significant decrease in connectivity between brain regions related to rumination and cognitive control. Taken together, these findings imply that CBT in general, and “Drop It” intervention in particular, may act by enhancing the top-down cognitive control of negative cognition or emotions (19).

A quite different interventional approach, transcranial direct current stimulation (tDCS), is one of the emerging noninvasive brain stimulation (NIBS) techniques that can also be used to alter RNT. During the application of tDCS, a weak, direct electric current is induced through anodal and cathodal scalp electrodes. Although the exact working mechanisms underlying tDCS are not yet fully understood, it is thought that tDCS exerts its beneficial effects through the induction of polarization shifts on the resting membrane potential (20). These alterations are considered sufficient to bias neural firing, with anodal stimulation locally facilitating cortical excitability and cathodal stimulation impairing it. To modulate cognitive performance and emotion regulation in healthy and neuropsychiatric subjects, the excitability-enhancing anodal electrode is most frequently applied to the DLPFC (21, 22). The cathodal electrode is often placed over a contralateral cephalic region such as the supraorbital region (21). Within the clinical context, the effect of tDCS on reducing rumination on sadness was demonstrated in patients with drug-resistant depression (23). Studies in healthy populations indicate that (even one) left anodal DLPFC tDCS session can attenuate momentary ruminative self-referential thoughts (24, 25) as well as self-attention (26). It is hypothesized that attenuated self-referential attention specifically may be a neurocognitive mechanism through which tDCS reduces emotional reactivity (26).

More recently, NIBS has been used together with cognitive/emotional tasks to increase the clinical effects. This approach is called online stimulation and is based on the activity-selectivity hypothesis (27), meaning that NIBS interventions may depend on the neural targets that are activated through cognitive tasks or therapies at the same time (28, 29). For instance, the combination of rTMS and psychotherapy in MDD yields higher remission rates than psychotherapy alone (30). Furthermore, as demonstrated by Brunoni and colleagues (31), the combination of tDCS and cognitive control therapy is beneficial for elderly depressed patients. Similarly, in a healthy population, the combination of a neuropsychological task with tDCS has been proven to more effectively improve specific cognitive functions (32, 33) such as counterfactual thinking (34). Therefore, activating, i.e., priming, target areas with RNT during active (as compared to sham) tDCS may yield higher benefits from the CBT.

Currently there are no studies that have evaluated the potential positive or negative effects of online tDCS priming combined with a CBT-based intervention on rumination. The aim of the present pilot study is to explore whether the combination of online active or sham tDCS priming, followed by group CBT “Drop It” treatment, is feasible and safe. Moreover, we hypothesized that priming rumination-related neurocircuits with an internal cognitive attention task focused on individual RNT combined with active (as compared to sham) tDCS prior to the group CBT “Drop It” sessions, would result in supplemental decreases of RNT in terms of reducing state rumination.

## Methods

### Participants

Eighteen participants (88% females), divided into two groups of nine participants each, participated in this study (*M* age = 44.8; *SD* = 8.9), however 17 were included in the final analysis as one participant was absent during more than two sessions (*n* active = 9). One of the inclusion criteria for participation in the study was that participants were already in mental health care treatment (psychiatrist, psychologist or general practitioner), seeking treatment for rumination - whether or not as part of a MDD or GAD diagnosis - and were referred by their treatment provider to “Drop It” (13, 35) – a psychoeducational CBT-based group intervention for RNT - at the Ghent University Hospital. As compensation for their participation in the study, participants were not charged for the “Drop It” intervention. All participants were between 32 and 60 years old. Habitual treatment use was allowed but kept at a steady dose during the entire experimental trial. Participants were excluded from the study in case of pregnancy, skin conditions in the skull area, use of implanted medical devices (such as a pacemaker), concentration difficulties, no motivation for weekly homework, no intention of weekly attendance, cognitive impairments, substance abuse, suicide risk, the diagnosis of obsessive–compulsive disorder or severe depression. All exclusion criteria were assessed by a psychiatrist during an intake interview using the Mini International Neuropsychiatric Interview [MINI (36)] as well as the Hamilton Depression Rating Scale [HDRS (37)]. The HDRS scores were collected as part of baseline measures and no participants were excluded based on cut-off scores. After the intake, participants were asked to fill in the Leuven Adaptation of the Rumination on Sadness Scale [LARSS (38)] and Penn State Worry Questionnaire [PSWQ (39)] at home. Before the experiment, the participants signed a written informed consent form. The study was approved by the Ethical Committee of the University Hospital of Ghent University (UZ Gent).

### Transcranial direct current stimulation

Stimulations were performed using a Soterix mini-CT tDCS device, which allows the double-blinding of the tDCS stimulation condition by providing individualized numeric codes. The anode was placed over the left DLPFC, located using the Beam F3 algorithm (40). Based on the distances between nasion, inion, tragus and vertex as landmarks, this algorithm estimates the coordinates for F3, resembling the left DLPFC (41). This area was selected based on our previous NIBS research in similar samples, targeting this exact same spot (29, 42). The cathode was placed on the right supraorbital region by placing the electrode 1 cm above the right eye. A current of 2 mA was delivered through carbon rubber electrodes of 4.5 × 4.5 cm that were covered by specially designed sponges soaked in a saline solution. During the active stimulation, there was a 30 s ramp-up period, followed by 20 min of stimulation, with a ramp-down of 30 s at the end. For sham tDCS, the current was directly ramped down after the initial ramp-up phase (43).

### Online tDCS

Online tDCS is defined here as the performance of an internal cognitive attention task focused on individual RNT concurrently with 20 min of stimulation. This task was adapted from the sixth session of the “Drop It” intervention (i.e., the mindfulness-based attention exercise, see the description of the “Drop It” intervention below). To guide this task, the patients listened in group to an audio recording. The following fragment is the transcript of the audio recording (translated from Dutch):

I want to ask you to visualize yourself in a situation which initiates worrying. What do you see? Where are you? What is happening? Who is involved? Which thoughts are running through your mind? Do you feel something in your body? How would you label these experiences? Are they associated with emotions? What would you call these emotions? What do you do? Stay with your attention to what you think and feel, no matter how annoying these thoughts or feelings are.

**Figure 1:**
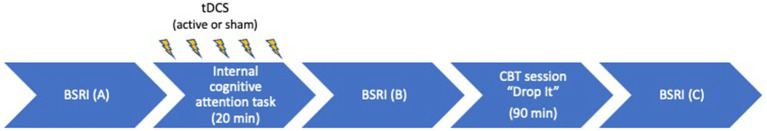
Overview of the study procedure. BSRI, Brief State Rumination Inventory; tDCS, transcranial direct current stimulation; CBT, cognitive behavioral therapy.

The audio recording started to play simultaneously with the start of the stimulation and lasted for 15 min. The last sentence (“*Stay with your attention …*”) was given at 15 min and as a consequence, for the last 5 min of the stimulation, the patients were instructed to stay with their feelings. Online tDCS was implemented before each weekly group CBT intervention “Drop It” (see “Drop It” and Figure 1).

Importantly, this online tDCS procedure acted as ‘primer’ for the subsequent CBT intervention “Drop It.”

### Drop It

The “Drop It” intervention consists of seven weekly group sessions (groups of up to 10 patients) and a follow-up session 1 month after the seventh session. All sessions lasted 90 min, were guided by CBT-trained psychotherapists and had a well-defined, following structure. All sessions started with a mindfulness-based attention exercise (15 min). Subsequently, a group discussion of the homework (15 min) followed by a “brain-talk” about relevant brain structures and neural circuits (15 min) took place. Lastly, a RNT exercise followed by a discussion (30 min) and a homework task (10 min) concluded the session. All participants received a manual for self-guided help explaining all exercises and homework tasks and a CD containing the attention training exercises. For a detailed description of the intervention see 13.

### Procedure

Half of the participants were randomized to receive active tDCS, whereas the other half received sham tDCS. Both groups attended the same CBT sessions to minimize group effects. To assure effective blinding, numeric codes were generated by the tDCS, where each code represented either active or sham stimulation. Every patient had a unique code assigned to them. Patients were required to enter the code in the tDCS device in order to start the stimulation. Patients and the psychotherapist were blinded for tDCS devices conditions until the end of the study. Stimulation was applied during the internal cognitive attention task focused on individual RNT. Subsequently, the tDCS setup was removed and the participants proceeded with the “Drop It” session as described above. During each session, participants were asked to fill in the Brief State Rumination Inventory [BSRI (44)] at three time points: before (A) and after (B) the stimulation as well as at the end of the “Drop It” session (C) (see Figure 1). Given that the first tDCS group CBT session was considered a practice session, these data were not included in the statistical analysis.

### Questionnaires

#### Hamilton depression rating scale

The HDRS is a standardized clinical interview developed to assess the severity of depression. Higher scores suggest higher levels of symptoms of depression (range 0–52). In the current study, the Dutch version of the 17-item HDRS was used (45). The calculated internal consistency was rather poor (Cronbach’s alpha = 0.57) in the current study. In general, the HDRS’ Cronbach’s alpha varies between 0.46 and 0.97 (46).

#### Leuven adaptation of the rumination on sadness scale

Leuven adaptation of the rumination on sadness scale is the Dutch version of the Rumination on Sadness Scale [RSS (47)]. It contains three original subscales from the RSS (minus four items) as well as eight new items. This questionnaire contains 21 items, which are scored on a 5-point Likert scale, ranging from 1 (‘totally not’) to 5 (‘very often’). Higher scores indicate higher levels of rumination (range 21–105). The internal consistency of the three subscales ‘Causal Analysis’, ‘Understanding,’ and ‘Uncontrollability’ is good to excellent, with Cronbach’s alpha of, respectively, 0.87, 0.85, and 0.91.

#### Penn state worry questionnaire

The PSWQ consists of 16 items that assess the general disposition to worry. Participants rate statements about worry on a scale of 1 (‘not at all typical of me’) to 5 (‘very typical of me’). Higher scores suggest a higher level of worry (range 16–80). In this study, the Dutch version of the questionnaire was used (48, 49) which has good internal consistency (Cronbach’s alpha between 0.83 and 0.86).

#### Brief state rumination inventory

The BSRI is a self-report questionnaire designed to measure RNT at the time of answering. The questionnaire consists of 8 items, each scored on a 100-mm VAS ranging from 0 (‘completely disagree’) to 100 (‘completely agree’). Higher scores indicate higher state rumination. In the current study, the Dutch version of the BSRI was used which has good internal consistency (Cronbach’s alpha = 0.89).

#### Tolerability and safety

Stimulation tolerability was assessed using a custom in-house developed questionnaire. At the end of each “Drop It” intervention, patients were asked to answer eight questions concerning transient hyperactivity or irritability, transient headache, transient local pain, transient neck pain, transient dental pain, transient tingling, transient changes in hearing, irritation at the site of stimulation. Patients could rate their experiences on a scale ranging from ‘never’ to ‘almost constantly’.

### Statistical analysis

#### Preprocessing

All preprocessing and analyses were performed using R (50) and MATLAB (MathWorks, Natick, MA). The data and the analysis code are publicly available at https://osf.io/4xtgr/.

One participant was excluded from further analysis due to absence during more than two sessions, resulting in 17 participants included in the final analysis. Subsequently, missing data was explored and visualized. Fifteen point 3% (15.3%) of the data was missing. Little’s Missing Completely at Random (MCAR) test was non-significant, χ^2^ = 173.37, *p* > 0.5, suggesting that the pattern of missing data was missing at random. Consequently, to handle missing values, a prediction model, i.e., multiple imputation method, using the ‘mice’ package (51) with default settings, was applied. The ‘mice’ function uses a Multivariate Imputation by Chained Equations (MICE) method to impute missing values. This method works by creating multiple imputed datasets and then pooling the results together. For sample characteristics, only complete questionnaires were considered. For the exploratory analysis using MATLAB, the amount of missing data for each participant was included as a one of the covariates. Finally, Δ (delta) rumination scores were calculated as the difference between BSRI scores at the end of the “Drop It” session and before the stimulation, i.e., C-A (*cf.* supra).

#### Analyses

Sample characteristics and potential group differences were explored using *independent sample t-tests* and *Fisher’s exact test*.

A mixed effects model was fitted using package ‘lme4’ (52) to investigate the relationship between Δ rumination scores (*M* = −13.24, *SD* = 286.24, range = −613 – 503) and the tDCS condition, CBT session as well as the interaction between the two. By-participant random intercepts were included to model individual differences with respect to rumination scores. Results of the model are reported using type III Wald chi-squared statistics. After fitting the model, the normality, linearity, homoscedasticity and multicollinearity assumptions were tested. No obvious violations were observed.

For the exploratory analysis, a one-way ANCOVA was used to determine whether Δ rumination scores, averaged across seven sessions, differed significantly between the active and the sham tDCS group. Age, gender, and the amount of missing data were included as covariates. A statistical significance level of *p* < 0.05, two-tailed, was adopted for all statistical tests.

## Results

### Sample characteristics

Participants were randomly assigned to an active tDCS (*n* = 9, 78% female, *M*_age_ = 48.56, SD = 8.49) or sham condition (*n* = 8, 100% female, *M*_age_ = 40.50, SD = 7.62). There were no significant baseline differences between the two conditions in terms of age [*t*(15) = 2.06, *p* = 0.06], gender (*p* = 0.47, *95%* CI *=* [0.001, 5.91]), HDRS scores [*t*(13.19) = −0.006, *p* = 0.99], LARSS scores [*t*(13.44) = 1.22, *p* = 0.25], PSWQ scores [*t*(11.69) = 0.43, *p* = 0.68], diagnosis (*p* = 0.33), pharmacological (*p* = 0.37) or psychological treatment (*p* = 1, *95%* CI *=* [0.07, 7.55]) (see Table 1). Patients were not able to correctly guess their assigned stimulation condition as the percentage of correct guesses was below chance (42%), indicating that blinding was effective. However, due to practical reasons, only a subset of participants was asked about guessing the stimulation condition.

**Table 1:** Baseline demographic and clinical characteristics of patients in the active versus sham tDCS condition.

Baseline characteristic	Active tDCS – CBT (*n* = 9)		Sham tDCS – CBT (*n* = 8)			
	*M*	*SD*	*M*	*SD*	*t*	*p*
Age (years)	48.56	8.49	40.50	7.62	2.06	0.06
HDRS	11.11 (9)	3.92	11.12 (8)	5.06	−0.006	0.99
LARSS	70.44 (9)	14.76	61.71 (7)	13.82	1.22	0.25
PSWQ	67.25 (8)	6.67	65.83 (6)	5.74	0.43	0.68
					95% CI	*p*
Female (%)	78		100		[0.001, 5.91]	0.47
Psychotherapy (%)					[0.07, 7.55]	1
Yes	55.56 (5)		62.50 (5)			
No psychotherapy	44.44 (4)		37.50 (3)			
Diagnosis (%)						0.33
MDD	33.33 (3)		37.50 (3)			
GAD	11.11 (1)		37.50 (3)			
Both	55.56 (5)		25.00 (2)			
Medication (%)						0.37
Antianxiety	0		12.50 (1)			
Antidepressant	22.22 (2)		50.00 (4)			
Both	44.44 (4)		12.50 (1)			
No medication	22.22 (2)		25.00 (2)			

CI, confidence interval; HDRS, the Hamilton Depression Rating Scale; LARSS, the Leuven Adaptation of the Rumination on Sadness Scale; PSWQ, the Penn State Worry Questionnaire. The number next to the mean of the three questionnaires is the sample size of each group after deleting the missing data.

### Rumination scores

**Figure 2:**
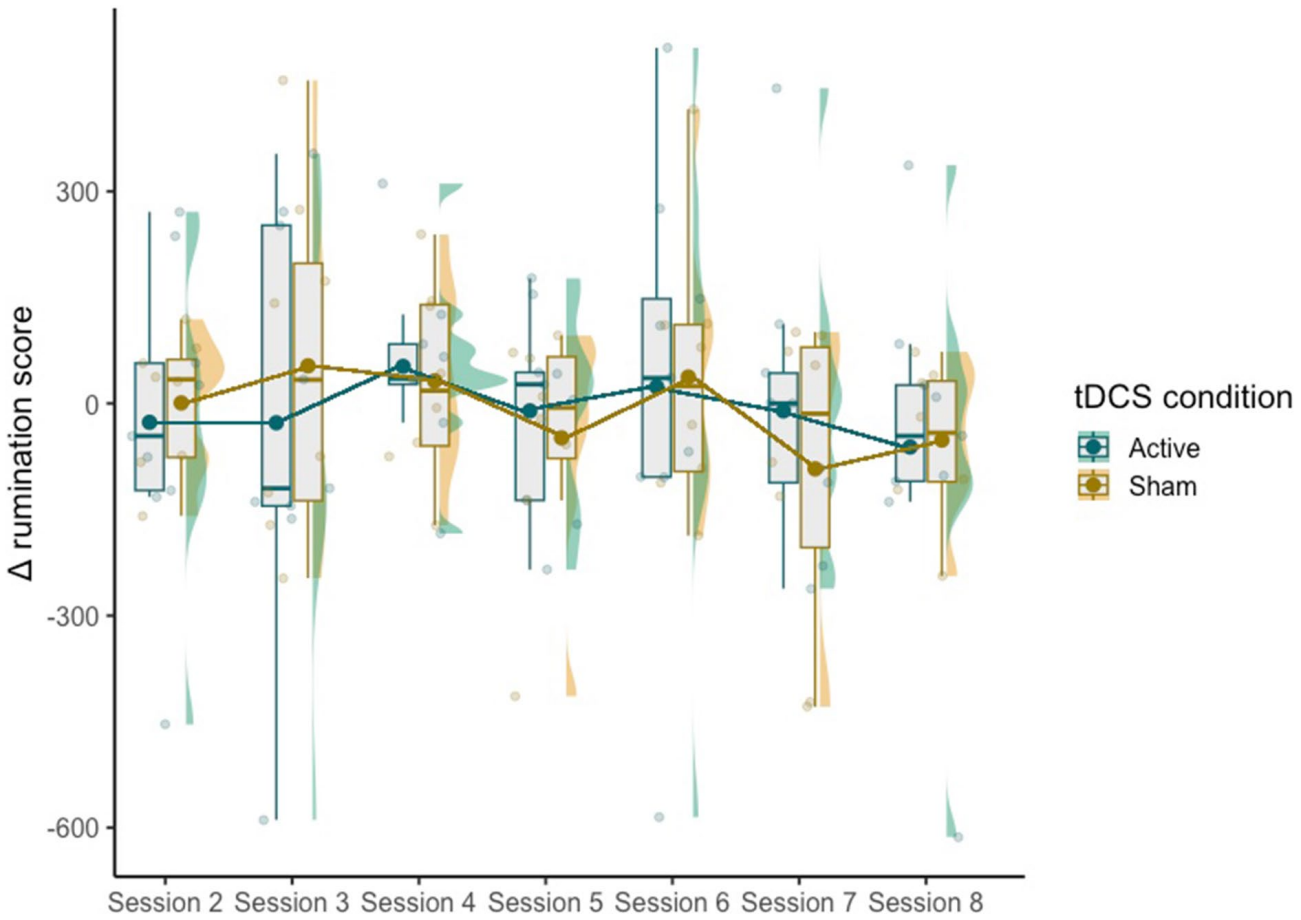
The distribution of the difference in post-CBT and pre-tDCS rumination scores.

The mixed effect model did not reveal significant effects of condition [*χ^2^*(1,115) = 0.19, *p* = 0.66], session [*χ^2^*(6,110) = 7.50, *p* = 0.28] or the interaction between the two [*χ^2^*(6,110) = 1.04, *p* = 0.98] on Δ rumination scores (Figure 2).

Additionally, Δ rumination scores were also calculated for the difference between BSRI scores after the stimulation and at the end of the “Drop It” session, i.e., B-A. However, no significant differences were found either [condition *χ^2^*(1,115) =0.05, *p* = 0.83], session [*χ^2^*(6,110) = 5.18, *p* = 0.52], interaction between the two [*χ^2^*(6,110) =2.07, *p* = 0.9].

### Exploratory analysis

**Figure 3:**
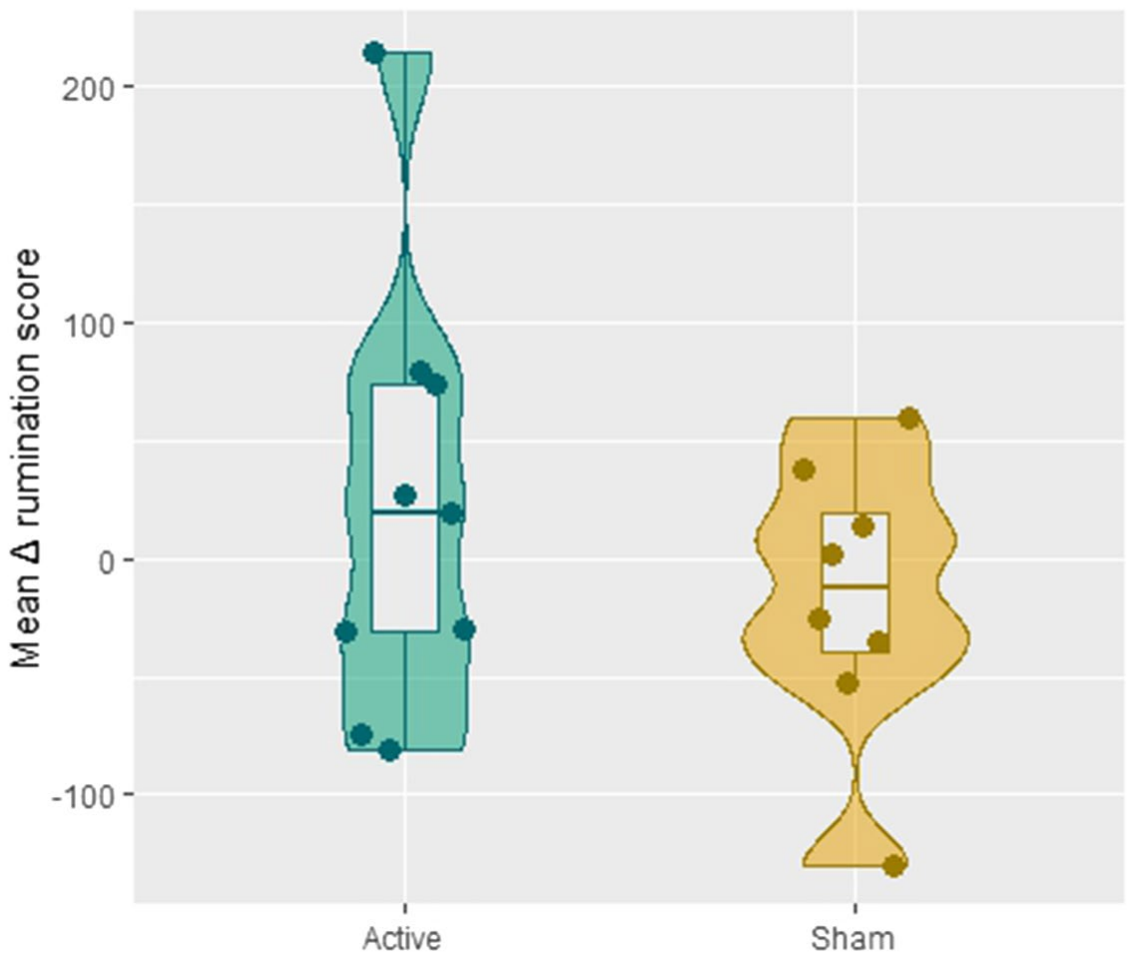
The difference in the averaged Δ rumination scores (i.e., the mean of the value C-A) across session 2–8 between the active and sham group.

The one-way ANCOVA revealed no significant difference in the averaged Δ rumination scores between the two groups [*F*(1,13) = 0.80, *p* = 0.38]: after receiving 8 sessions of CBT, patients in the active tDCS group (*M* = 21.89, SD = 92.49) did not ruminate less compared with the sham group (*M* = −16.43, SD = 58.97; see Figure 3).

### Tolerability and safety

No severe short-term adverse effects were reported during the study. Long-term effects were not assessed. Participants mostly reported irritation at the site of stimulation, transient tingling, and transient headache, which is in line with previously described adverse effects of tDCS (53). Due to practical reasons, only a subset of patients was asked to fill in our tolerability questionnaire. However, given the preliminary results as well as just one drop out throughout the whole study, it seems reasonable to deduce that the combination of group CBT and tDCS was well tolerated.

## Discussion

In this pilot study, we have evaluated a novel therapeutic approach aimed at reducing rumination by combining an internal cognitive attention task focused on individual RNT and tDCS, i.e., online tDCS priming, with a CBT-based group intervention “Drop It.” A sham-controlled online tDCS priming followed by CBT-based group intervention was conducted in 17 depressed and/or anxious patients who mainly suffered from RNT. Statistical analysis showed no significant additional effect of online tDCS priming with group CBT on state rumination. Given no reports of severe adverse effects and just one drop out, the combination of group CBT and tDCS seems feasible, well-tolerated and safe. The minor adverse effects reported by the participants pertained to transient events and were fully in line with previously described adverse effects of tDCS. Although we were unable to detect significant differences in state rumination scores between the tDCS groups, our current findings indicate that focusing on RNT during tDCS does not negatively impact (group) CBT.

Besides the relatively small sample size, the lack of additional effects of online tDCS priming is puzzling. Firstly, it could be that the amount of primed tDCS sessions was critically too low (seven tDCS sessions over 7 weeks time and one follow-up session). Martin and colleagues (54) examined the efficacy of tDCS combined with cognitive emotional training and demonstrated significant antidepressant efficacy. However, tDCS administered during cognitive emotional training was applied three times a week for 6 weeks. In a group of alcohol dependent patients, Dubuson and colleagues (55) found that five consecutive daily sessions for 4 weeks combined with alcohol cue inhibitory control training resulted in better clinical outcomes. Although it is difficult to directly compare studies due to methodological differences, it could be that one online tDCS priming before CBT, applied on a weekly basis, is simply not sufficient to elicit meaningful clinical differences. Similarly, although the choice of the stimulation zones in the current study (anodal left prefrontal - cathodal right supraorbital) was based on our previous research (29, 42), it could be that in the present context they were less suitable. Secondly, it is possible that using a cognitive attention task focused on individual RNT to prime the neural targets might not have been the most optimal choice. As argued before, we expected that priming rumination-related neurocircuits before CBT with active online tDCS would result in a supplemental decrease of rumination. However, notwithstanding that we could not demonstrate significance, our exploratory analysis – contrasting active and sham primed stimulation over the seven group CBT sessions – is suggestive that active online tDCS priming may result in rumination increments after CBT. It remains an open question regarding the kind of cognitive tasks used during the tDCS stimulation. For instance, Sreeraj and colleagues (56) evaluated the effect of a single session online tDCS on working memory in schizophrenia and found improved working memory performance only in the online sham condition. Moreover, an open question remains regarding the timing of the tDCS/CBT combination that consists of multiple sessions, e.g., before versus immediately after the CBT. Lastly, other negative results of combining tDCS with therapeutic interventions have been documented. For example, tDCS-enhanced inhibitory control training showed no superior efficacy on symptoms of PTSD, anxiety, or depression (57).

## Limitations

Besides the relatively small sample size, we did not include clinical post-measurements. Therefore, we cannot claim that the combined active versus sham tDCS priming group CBT intervention yielded additional clinical effects on RNT in these patients. While online tDCS priming did not seem to have an augmenting effect on our primary outcome, being state rumination, we cannot rule out that it still had an effect on the trait of worry and/or rumination. This limits our conclusions about potential beneficial clinical effects of tDCS and all interpretations should be restricted to the state effect of our combined intervention on rumination. Moreover, as the first tDCS group CBT session was considered a practice session, this data was not included in the statistical analysis. However, active and sham stimulation was nevertheless applied, which could have influenced the outcomes of the following sessions. The goal of the practice session was to familiarize the patients with the devices and the structure of the sessions. However, in future studies, when including a practice session, one could consider using the tDCS devices but not turning them on, in order to prevent possible impact on the targeted neural areas. Additionally, the lack of a control condition, e.g., tDCS alone or CBT alone, limits our conclusions about the tDCS intervention.

Furthermore, we used a custom in-house developed questionnaire to assess tolerability. In future studies a standardized questionnaire should be used to assure reliable responses as well as comparability to established norms and/or other studies. Additionally, the use of only a subset of the total sample size (due to practical reasons) limited statistical options of analyzing side-effects to quantify tolerability. Similarly, asking all participants about guessing the stimulation condition, instead of only a subset, should be assured in future studies.

Finally, besides the idea that other stimulation methods, such as rTMS, could have been more appropriate, it could also be informative to examine specific cognitive subtypes of rumination, i.e., brooding and reflection. For instance, a recent rTMS study by Ehrlich and colleagues (58) showed that repetitive pulse transcranial magnetic stimulation may modulate reflection rumination rather than brooding. Additionally, an interesting approach for future studies could be to explore the influence of tDCS on more objective measures of rumination, such as cardiac activity. More specifically, both in healthy and clinical populations, it has been demonstrated that heart rate variability (HRV) is negatively correlated with rumination, such that lower levels of HRV indicate higher levels of rumination (59, 60). In the context of NIBS, the combination of tDCS and cardiac biofeedback has proven to be effective in reducing psychological and physiological stress responses (61).

## Conclusion

This is the first study to explore the potential positive or negative effects of online tDCS priming combined with an 8-week group CBT-based intervention “Drop It” on state rumination. Although the experimental protocol was found to be safe, well-tolerated and feasible, we could not demonstrate superior efficacy of tDCS-augmented CBT on RNT. Future well-powered RCTs may be needed to demonstrate additional clinical effects of online tDCS priming on psychotherapeutic interventions, exploring other types of cognitive tasks paired with tDCS as well as more objective neurophysiological measurements. Additionally, it could prove to be mandatory to augment the number of primed tDCS sessions during the period patients receive (group) CBT.

## Data availability statement

The datasets presented in this study can be found in online repositories. The names of the repository/repositories and accession number(s) can be found at: OSF repository https://osf.io/4xtgr.

## Ethics statement

The studies involving human participants were reviewed and approved by Ethical Committee of the University Hospital of Ghent University (UZ Gent). The patients/participants provided their written informed consent to participate in this study.

## Author contributions

JR, RD, M-AV, GL, and CB participated in the conception and design of this study. SDW and SDS were involved in data acquisition. PH, SDS, CW, and G-RW undertook the data preprocessing and statistical analysis. PH, CW, SDW, SDS, M-AV, G-RW, and CB participated in data interpretation and in writing the manuscript. All authors contributed to the article and approved the submitted version.

## Funding

The authors acknowledge the following funding. G-RW was supported by the National Natural Science Foundation of China (Grant No. 62271415). CW was supported by fellowship funding from the China Scholarship Council (CSC). SDS was funded by a FWO-Flanders PhD fellowship (Grant Number: 11J7521N). This work was also supported by the Queen Elisabeth Medical Foundation for Neurosciences; by the Ghent University Multidisciplinary Research Partnership “The integrative neuroscience of behavioral control”; by an Applied Biomedical (TBM) grant of the Agency for Innovation through Science and Technology (IWT); part of the Research Foundation – Flanders (FWO) PrevenD Project 2.0 (T000720N) and FWO project G011018N. M-AV received funding from the FWO and from Ghent University (Grants: G0F4619N and BOF17/STA/030, respectively).

## Conflict of interest

The authors declare that the research was conducted in the absence of any commercial or financial relationships that could be construed as a potential conflict of interest.

## Publisher’s note

All claims expressed in this article are solely those of the authors and do not necessarily represent those of their affiliated organizations, or those of the publisher, the editors and the reviewers. Any product that may be evaluated in this article, or claim that may be made by its manufacturer, is not guaranteed or endorsed by the publisher.
